# Free‐breathing conformal irradiation of pancreatic cancer

**DOI:** 10.1120/jacmp.v14i4.4152

**Published:** 2013-07-08

**Authors:** Ignazio Solla, Sergio Zucca, Marco Possanzini, Sara Piras, Claudio Pusceddu, Sergio Porru, Gianfranco Meleddu, Paolo Farace

**Affiliations:** ^1^ Department of Radio‐Oncology Regional Oncological Hospital Cagliari Italy

**Keywords:** four‐dimensional CT, pancreas, radiotherapy, CBCT, intrafractional motion, interfractional motion

## Abstract

The purpose of this study was to assess treatment margins in free‐breathing irradiation of pancreatic cancer after bone alignment, and evaluate their impact on conformal radiotherapy. Fifteen patients with adenocarcinoma of the head of the pancreas underwent implantation of single fiducial marker. Intrafraction uncertainties were assessed on simulation four‐dimensional computed tomography (4D CT) by calculating maximal intrafraction fiducial excursion (MIFE). In the first ten patients, after bony alignment, the position of the fiducial was identified on weekly acquired megavolt cone‐beam CT (MV‐CBCT). The interfraction residual uncertainties were estimated by measuring the fiducial displacements with respect to the position in the first session. Patient mean (pM) and patient standard deviation (pSD) of fiducial displacement, mean (μM) and standard deviation (μSD) of pM, and root‐mean‐square of pSD ($\sigma_{\text{res}}$) were calculated. In the other five patients, MIFE was added to the residual component to obtain personalized margin. In these patients, conformal kidney sparing (CONKISS) irradiation was planned prescribing 54/45 Gy to PTV1/PTV2. The organ‐at‐risk limits were set according to current NCCN recommendation. No morbidity related to the fiducial marker implantation was recorded. In the first ten patients, along right–left, anterior–posterior, and inferior–superior directions, MIFE was variable (mean±std=0.24±0.13cm,0.31±0.14cm,0.83±0.35cm, respectively) and was at most 0.51, 0.53, and 1.56 cm, respectively. Along the same directions, μM were 0.09,−0.05,−0.05cm,μSD were 0.30, 0.17, 0.33 cm, and $\sigma_{\text{res}}$ were 0.35, 0.26, and 0.30 cm, respectively. MIFE was not correlated with pM and pSD. In the five additional patients, it was possible to satisfy recommended dose limits, with the exception of slightly higher doses to small bowel. After bony alignment, the margins for target expansion can be obtained by adding personalized MIFE to the residual interfraction term. Using these margins, conformal free‐breathing irradiation is a reliable option for the treatment of pancreatic cancer.

PACS number: 87.55.D‐

## I. INTRODUCTION

Radiation therapy has been an important option in the treatment of patients affected with locally advanced or recurrent pancreatic carcinoma, both in the postoperative and in the preoperative setting. It is conventionally delivered by three‐dimensional conformal or intensity‐modulated external beams, using alignment to bony anatomy for patient positioning. Given the presence of critical organs around the pancreas, such as the duodenum, beam overshoot or undershoot due to respiratory motion may increase patient risk. To account for interfractional and intrafractional variations, a planning target volume (PTV) is typically added to cover the clinical target volume. The causes of interfractional changes are daily variations in organ fillings (stomach and bowel), tumor size, patient weight, and treatment‐induced tissue changes, whereas intrafractional motion variations may be caused by peristalsis, cardiac motion, and respiration.

Recently, the effect of intrafractional organ movement throughout the breathing cycles has been investigated for the treatment of pancreatic cancer. Intrafractional motion due to respiration can be determined by four‐dimensional computed tomography (4D CT) that permits reconstruction of three‐dimensional CT scans at various phases of the respiratory cycle. The PTV can be constructed using respiratory correlated 4D CT^1^, ^2^, ^3^, ^4^ or CINE‐MRI^(5)^ to determine acceptable treatment margins.

However, there is substantial residual uncertainty using respiratory gating and patient positioning based purely on bony anatomy. In Jayachandran et al.,^(6)^ respiratory‐gated portal images showed that bony anatomy matched tumor position in only 20% of the radiation treatments. Interfraction residual errors can be due to bowel gas or daily changes in the position of surrounding normal tissue, daily baseline diaphragm position, or day‐to‐day variation in the breathing pattern.

Cone‐beam CT (CBCT) has been increasingly used in the image‐guided setting.^7^, ^8^ The effect of moving volumes under free‐breathing CBCT has been investigated,^(9)^ and CBCT projection were recently used to assess inter‐ and intrafraction motion of the pancreas.^(10)^

The present study aims to provide further data on the residual uncertainty after alignment to bony anatomy, and to compare such interfraction uncertainty with intrafractional motion. For this purpose, implanted fiducial seeds were used as a surrogate for tumor and pancreatic head location. Respiration intrafraction motion was analyzed by 4D CT acquired during simulation, and residual interfractional motion was analyzed by megavolt cone‐beam CT (MV‐CBCT) acquired during the treatment course of ten patients. Finally, to verify whether the corresponding margin allows 3D conformal irradiation within recommended limits, five additional patients were planned by a conformal kidney sparing (CONKISS) technique.

## II. MATERIALS AND METHODS

### A. Design of the study

Fifteen consecutive patients affected by locally advanced nonoperable adenocarcinoma of the head of the pancreas underwent implantation of a single fiducial marker. In all patients, intrafractional motion was assessed by 4D CT. In the first ten patients, after bony alignment, the position of the fiducial was identified on weekly acquired MV‐CBCT. The interfraction residual uncertainties were estimated by measuring the fiducial displacements with respect to the position on the MV‐CBCT acquired in the first session. In the successive five patients, personalized PTVs margins were calculated adding the intrafractional margin to the residual interfraction component assessed on the first ten patients. Conformal free‐breathing irradiation was then planned to verify whether, with these margins, it is possible to fulfill the limits recommended by the National Comprehensive Cancer Network (NCCN) Guidelines (version 2.2012).

### B. Fiducials implantation and patient preparation

In each patient, a single golden solid fiducial marker (CIVCO Medical Solution, Orange City, IA), with diameter=1.2mm and length=3mm was implanted. Every patient gave the necessary informed consent to the fiducial implantation. In each patient, the coagulation function was verified before fiducial marker implantation and the blood count was verified before and after the procedure, developed in day hospital regimen. To allow for possible drift of the fiducial, which may cause deviation, we waited at least seven days between implantation and the acquisition of 4D CT, as previously suggested.^(6)^ Treatment preparation and execution were performed in supine position, using dedicated immobilization device. The day before the acquisition of simulation 4D CT, each patient was trained to breathe in a regular way. To control, at least partially, the part of the organ movement due to the visceral motion, patients were asked to fast before the simulation CT and before each treatment session.

### C. Intrafractional motion assessment by 4D CT

Simulation CT (Sensation Open, Siemens, Concorde, CA) was acquired in 4D mode, using a spiral protocol with small (0.1) pitch factor. The spiral CT data were acquired in conjunction with the acquisition of respiratory waveforms by a sensor (Anzai Medical, Tokyo, Japan) detecting pressure changes due to abdominal motion. The respiratory level (amplitude) is optimized by an adjustment regarding gain, and offset to display inspiration maximum at 100% and expiration minimum at 0% of signal amplitude. Three‐dimensional CTs were retrospectively reconstructed (pixel size=0.8×0.8mm;slice thickness=2mm) at the following eight amplitudes of the respiratory waveforms: 25%, 50%, 75%, and 100% of inspiration (IN), and 75%, 50% 25%, and 0% of expiration (EX). The positions of the fiducial marker in each of the eight phases were measured. The maximal intrafraction fiducial excursion (MIFE) was assessed in each patient along right‐left (RL), anterior‐posterior (AP), and inferior‐superior (IS) directions.

### D. Interfractional residual errors assessment by MV‐CBCT

Megavolt cone‐beam CT (MV‐CBCT) was acquired on Siemens ONCOR Impression^PLUS^ system (Siemens, Malvern, PA) for patient positioning before treatment sessions, using a flat panel of silicon detectors ($\text{OPTIVUE}\;1000\text{ST}\;1024\times 1024\;\text{pixels}-40\times 40\;{\text{cm}}^{2}$) (Siemens). All measurements were performed by means of MVision 2.0 package (Siemens), and image acquisition covered an arch of 200° (from −90° to 110°) by a 6 MV beam, using a protocol with nine monitor units. A total of 200 projections were sampled by the flat panel during the rotation. Images were reconstructed by filtered back‐projection, with a matrix size of 256×256, covering a maximum field view of $27\times 27\;{\text{cm}}^{2}$. The duration of MV‐CBCT (about 50 sec) covers many respiratory cycles (typically 3–5 sec long).

Since, due to respiration motion, the fiducials appeared blurred, their positions were identified as the voxel of maximal intensity. In each patient, to assess residual errors after alignment on bony landmarks, the positions of the fiducial in each treatment session were compared with the corresponding position in the MV‐CBCT acquired in the first session.

For each patient, the mean (pM) and the standard deviation (pSD) of fiducial displacement were calculated along RL, AP, and IS directions. Then, the mean (μM) and the standard deviation (μSD) of the patient population pM were estimated. The root mean square of pSD ($\sigma_{\text{res}}$) was used as the best estimation for the execution component of the residual error.

Finally, the correlation between pM, pSD, and MIFE were assessed along any direction by the Pearson test (*r*).

### E. CTV to PTV margin estimation

An anisotropic CTV to PTV margin of 1 cm along RL and AP and 2 cm along IS was preliminary added to CTV in order to obtain PTV. These margins were retrospectively compared with those according to van Herk:^(11)^

(1) $${\left[CTV-PTV\right]}_{fiducial}=2.5\sqrt{\sum_{bone}^{2}+\sum_{res}^{2}\;}+1.64\sqrt{\sigma_{bone}^{2}+\sigma_{res}^{2}-\sigma_{p}^{2}}-1.64\sigma_{p}^{2}+\frac{MIFE}{2}$$

where $\Sigma_{\text{bone}},\Sigma_{\text{res}},\Sigma_{\text{bone}}$, and $\Sigma_{\text{res}}$ are the bone/residual component of the preparation/execution setup errors, and $\Sigma_{p}$ denotes the SD of the dose gradient or “penumbra” for which a value of 3.2 mm was used. $\Sigma_{\text{bone}}$ and $\Sigma_{\text{bone}}$ were assigned a 2 mm value, as the residual error after bone alignment is related to the positioning of the flat panel which was characterized by an accuracy of 2 mm.^(12)^ To take into account respiratory motion, when the midrespiratory position is known, a linear addition of half peak‐to‐peak motion amplitude has been suggested.^10^, ^13^ Accordingly, half of the MIFE was symmetrically added to the fiducial setup margin to allow a direct comparison with the results reported by other studies.

### F. Treatment planning and evaluation

Treatment planning was performed on the CT acquired at 0% of expiration, where the MIFE component was asymmetrically applied to the CTV to obtain the PTV. A five‐beam arrangement was applied, as described in the 3D conformal CONKISS method.^(14)^ This method uses one anterior–posterior‐like beam with 40° gantry (G40) and 90° table angle (T90) and four lateral fields, G270–T340, G90–T340, G270–T20, and G90–T20. The gantry angles of lateral fields and the table angle of the anterior–posterior‐like beam were adjusted so that, from their beam's eye view (BEV), the same kidneys areas were overlapped in the PTV. For the four lateral beams, a 60° wedge was used. To further increase PTV homogeneity and reduce the maximum dose value, a second segment was used in the anterior–posterior‐like beam that excluded the highest 2%–3% dose.

Dose prescription was 45 Gy to the locoregional lynphonodes (PTV2), plus a sequential 9 Gy boost to the macroscopic disease volume (PTV1), planned by the same beam arrangement. Beam weights were calculated by inverse planning using the following objectives: kidney $V_{18\text{Gy}}<30\%$, stomach max. <55Gy; small bowel max. dose<55Gy, liver mean dose<30Gy, and spinal cord max. dose<45Gy. PTV coverage and the corresponding dose to the organs at risk were calculated, and compared with the limits recommended by NCCN guidelines.

## III. RESULTS

With the exception of mild abdominal pain in the implantation site (G1–G2, according to NCI Common Terminology Criteria for Adverse Events – CTCAE v.4) requiring common anti‐inflammatory drugs, no other morbidity related to the fiducial marker implantation was recorded.

**Figure 1:**
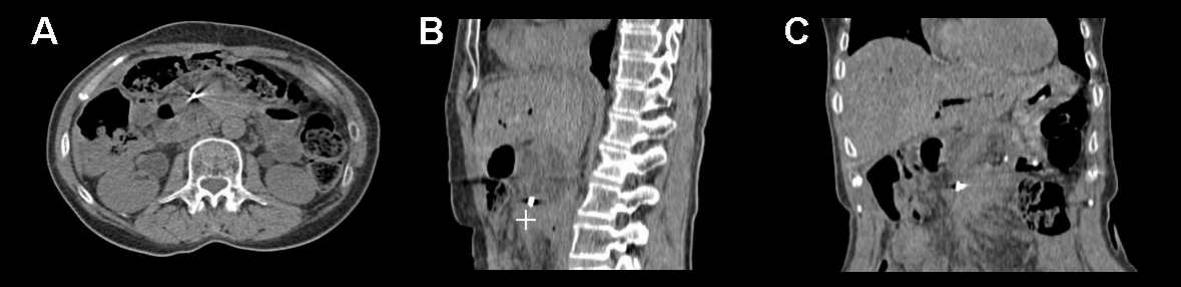
Axial (a), sagittal (b), and coronal (c) views of patient #10 at 0% expiration, acquired by 4D CT. The fiducials were always clearly distinguishable. The corresponding position of the fiducial at 100% inhalation is also shown (symbol ‘+’) in the sagittal section.

The fifteen patients were able to breathe regularly both in the training session and during the CT acquisition and were therefore all enrolled in the study. The fiducials were clearly visible in CT scan (Fig. 1). They moved in a varying orbit during the respiratory cycle in a hysteresis‐like motion. The measured maximal displacements (MIFE) are reported in Table 1 for each patient. MIFE was variable among patients (mean±std=0.24±0.13cm,0.31±0.14cm, and 0.83±0.35cm) and was at most 0.51, 0.53, and 1.56 cm, along RL, AP, and IS directions, respectively.

**Figure 2:**
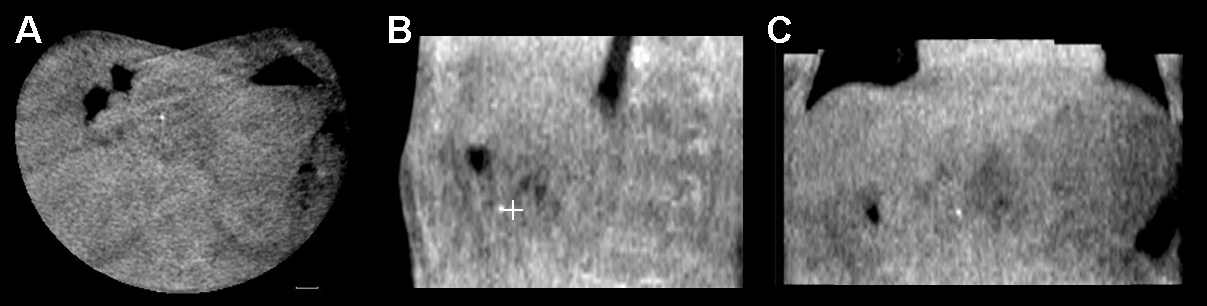
Axial (a), sagittal (b), and coronal (c) views of MV‐CBCT for one treatment session in patient #6. The fiducial appeared as a single blurred point. The position of the fiducial in the first MV‐CBCT is also shown (symbol ‘+’) in the sagittal section.

On MV‐CBCT, the fiducials appeared blurred (Fig. 2). A total of 61 MV‐CBCT, acquired during the treatments of the first ten patients, were analyzed. After alignment to bony anatomy, the positions of the fiducial in the MV‐CBCT were compared with the positions in the first

**Table 1:** Intrafraction respiratory fiducial excursion by 4D CT

	*MIFE (cm)* ^*a*^		
	*RL*	*AP*	*IS*
# 1	0.51	0.42	1.01
# 2	0.41	0.27	0.60
# 3	0.24	0.20	1.00
# 4	0.18	0.08	0.27
# 5	0.17	0.32	0.80
# 6	0.23	0.46	0.60
# 7	0.17	0.16	0.80
# 8	0.09	0.28	0.60
# 9	0.24	0.53	1.01
# 10	0.17	0.36	1.56
# 11	0.23	0.30	0.96
# 12	0.45	1.05	1.40
# 13	0.07	0.16	0.38
# 14	0.07	0.10	0.23
# 15	0.20	0.40	1.51
Mean	0.23	0.34	0.85
Std	0.13	0.24	0.42

a Maximal intrafraction fiducial excursion (MIFE) in the fifteen patients (Nos. 1–15) along RL, AP, and IS directions.

**Figure 3:**
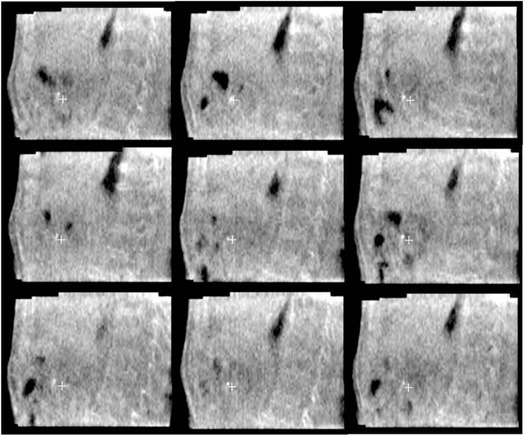
Sagittal views of MV‐CBCT for nine different treatment sessions in patient #8. The position of the fiducial in the first MV‐CBCT is shown (symbol ‘+’).

MV‐CBCT (see some examples in Fig. 3). The residual shifts of the fiducial were measured along the three main axes (Table 2). The absolute maximum shifts in the RL, AP, and IS directions were 1.51 cm, 0.93 cm, and 1.05 cm, respectively. The measured pM, pSD, μM,μSD, and $\Sigma_{\text{res}}$ are also reported along LR, AP, and IS directions: μM were 0.09,−0.05, and $0.05\;\text{cm};\mu_{\text{SD}}$ were 0.30, 0.17, and 0.33 cm; and $\Sigma_{\text{res}}$ were 0.35, 0.26, and 0.30 cm, respectively.

The MIFE displacements did not significantly correlate (Pearson's r test) with both residual pM and pSD along any directions, satisfying requirements for direct linear addition of MIFE motion margin to setup margin and thus the application of Eq. (1).

A posteriori, the described CTV to PTV margin recipe was applied on the additional five patients (#11–#15) using the MIFE values reported in Table 1. The obtained margins are reported in Table 3. $\Sigma_{\text{bone}}$ and $\Sigma_{\text{bone}}$ were assigned a 2 mm value, as described in the Materials & Methods section above. The standard deviation (pSD) of fiducial displacement with respect to the first MV‐CBCT (assessed in the first ten patients and reported in Table 2) does not fully represent the preparation component of the residual error $X_{\text{res}}$, which should be evaluated with respect to the CT simulation. Therefore the root mean square of pSD ($\Sigma_{\text{res}}$) was used as the best estimation of X in Table 3. For comparison, in Table 3 we report also the margins recently calculated by other authors.

Finally in Table 4, the results of the dosimetric study are reported. Both PTV1s and PTV2s coverage was always satisfactory (>95%). Only the stomach and small bowel slightly exceed the recommended values. However, the volumes exceeding 55 Gy were limited. In patients #11 and #15 only, the small bowel $V_{55\text{Gy}}$ were around 4 and 12 cc, respectively. The dose delivered to the other organs at risk were largely below the recommended limits, with the exception of ipsilateral kidney in patient #12. In the same patient, the $V_{18\text{Gy}}$ of combined kidney was 2% higher than the recommended value.

**Table 2:** Interfraction residual shifts by MV‐CBCT.^a^

	*RL (cm)*	*AP (cm)*	*IS (cm)*
# 1^b^	0.64±0.48	−0.05±0.11	−0.35±0.29
# 2	0.05±0.08	0.08±0.07	0.25±0.19
# 3	0.05±0.38	0.18±0.40	−0.04±0.44
# 4	−0.16±0.33	0.05±0.02	−0.38±0.14
# 5	0.02±0.19	−0.18±0.20	−0.37±0.35
# 6	0.02±0.34	−0.09±0.20	0.09±0.31
# 7	0.59±0.55	−0.43±0.23	0.01±0.15
# 8	−0.19±0.26	−0.07±0.18	0.40±0.20
# 9	−0.12±0.20	−0.10±0.50	0.59±0.41
# 10	0.24±0.20	0.09±0.05	−0.19±0.14
μM	0.09	−0.05	−0.05
μSD	0.30	0.17	0.33
$\Sigma_{\text{res}}$	0.35	0.26	0.30

a Shifts of the fiducial on treatment MV‐CBCT with respect to the first MV‐CBCT along the right–left (RL), anterior–posterior (AP), and inferior–superior (IS) directions.

b For patients Nos. 1–10: patient mean(pM)±patient standard deviation(pSD) of the residual errors.

μM=population mean

μSD= standard deviation of the patient population

$\Sigma_{\text{res}}=\text{root mean square of pSD}$

**Table 3:** CTV to PTV margins

		*RL (cm)*	*AP (cm)*	*IS (cm)*
Setup error bony anatomy	Systematic component ($\Sigma_{\text{bone}}$)^b^	0.20	0.20	0.20
	Random component ($\Sigma_{\text{bone}}$)^b^	0.20	0.20	0.20
Residual error	Systematic component ($\Sigma_{\text{res}}$)^c^	0.35	0.26	0.30
	Random component ($\Sigma_{\text{res}}$)	0.35	0.26	0.30
CTV to ITV MARGINS	individual MIFE/2	see Table 1	see Table 1	see Table 1
CTV to PTV MARGINS	Patient # 11	1.26	1.14	1.54
	Patient # 12	1.37	1.51	1.76
	Patient # 13	1.18	1.07	1.25
	Patient # 14	1.19	1.04	1.17
	Patient # 15	1.25	1.19	1.81
*CTV to PTV MARGINS (from Whitfield et al*.^*(10)*^ *)* ^*a*^		*1.20*	*1.20*	*2.30*

a CTV to PTV margins calculated using Eq. (1) in five patients (Nos. 11–15). The residual errors were calculated in the first ten patients (Nos. 1–10).

b Equal to the accuracy of flat panel positioning.

c Equal to $\sigma_{\text{res}}$.

**Table 4:** Dosimetric parameters

*Contour*	*Parameter (units)*	*#11*	*#12*	*#13*	*#14*	*#15*	mean±std
PTV1	Volume (cc)	371.0	369.7	376.6	228.7	407.6	350.7±70.0
	$V_{95\%}(\%)$ ^b^	97.5	95.4	96.5	98.9	99.5	97.5±1.7
CTV1	Volume (cc)	92.3	71.7	99.2	65.1	137.9	93.2±28.6
	$V_{95\%}(\%)$	100.0	100.0	100.0	100.0	100.0	100.0±0.0
PTV2^a^	Volume (cc)	558.2	900.4	602.9	551.7	907.6	704.2±183.5
	$V_{95\%}(\%)$	95.2	95.0	95.2	95.7	95.0	95.2±0.3
CTV2^a^	Volume (cc)	141.9	190.1	173.4	191.6	262.5	191.9±44.2
	$V_{95\%}(\%)$	100.0	99.9	99.9	99.9	100.0	99.9±0.1
Liver	$D_{\text{mean}}(\text{Gy})$	26.5	22.6	23.1	21.4	23.1	23.3±1.9
Spleen	$D_{\text{mean}}(\text{Gy})$	9.2	8.7	7.1	20.9	11.1	11.4±5.5
Spinal Cord	$D_{\text{max}}(\text{Gy})$	15.0	15.6	14.0	13.2	16.7	14.9±1.4
	Volume (cc)	174.9	223.6	255.2	227.5	257.8	227.8±33.4
Stomach	$D_{\text{max}}(\text{Gy})$	56.2	55.2	55.4	56.0	54.2	55.4±0.8
	$V_{55\text{Gy}}(\text{cc})$ ^c^	2.11	0.03	0.81	0.85	0.00	0.76±0.86
	Volume (cc)	581.8	406.2	340.1	516.7	389.7	446.9±99.3
Small Bowel	$D_{\text{max}}(\text{Gy})$	55.5	55.7	55.7	55.9	56.1	55.8±0.2
	$V_{55\text{Gy}}(\text{cc})$	4.32	1.25	2.19	3.71	12.27	4.75±4.38
Right Kidney	$D_{\text{mean}}(\text{Gy})$	8.9	21.6	7.8	8.0	14.2	12.1±5.9
	$V_{18\text{Gy}}(\%)$ ^c^	10.9	41.5	7.0	12.0	20.5	18.4±13.8
Lift Kidney	$D_{\text{mean}}(\text{Gy})$	7.4	14.1	4.4	7.2	12.5	9.1±4.0
	$V_{18\text{Gy}}(\%)$	8.6	25.2	1.5	10.7	13.0	11.8±8.7
Combined Kidneys	$V_{18\text{Gy}}(\%)$	9.7	32.0	3.9	11.4	16.6	14.7±10.7

a $V_{95\%}$ of PTV2 and CTV2 were calculated without boost prescription.

b $V_{95\%}=$ percentage of volume receiving at least 95% of the prescribed dose.

c $V_{55\text{Gy}}$, and $V_{18\text{Gy}}=$ percentage of volume receiving more than 55 Gy 18 Gy, respectively.

$D_{\text{mean}}=\text{mean dose}$

$D_{\text{max}}=\text{max dose}$

## IV. DISCUSSION

In the last years, the respiratory motion of the pancreas was described using different techniques. Recently, 4D CT was used to quantify pancreatic motion,^1^, ^2^, ^3^, ^4^ revealing an hysteresis‐like motion and supporting the use of individual anisotropic expansion, mainly in the IS direction, to define the PTV. Our findings confirmed that in the treatment of pancreatic cancer, the largest motion component was in the IS direction and that personalized anisotropic margin should be used to account for intrafraction respiratory motion in nongated treatment.

Using fiducials implanted in tumors is not commonly practiced, since this involves an invasive, potentially risky procedure, and only a few studies have used fiducial markers in the pancreas to report intrafractional movements.^10^, ^15^, ^16^ In our study, no morbidity related to the fiducial marker implantation was recorded.

Whereas the advent of 4D CT has increased the data available on intrafraction respiratory motion, fewer studies documented interfractional motion in pancreatic cancer. One study reported substantial interfractional changes in three patients.^(17)^ Another study assessed interfractional breath‐hold reproducibility in ten patients.^(18)^ Recently, repeated 4D CT was applied to fifteen patients, using intrapancreatic bile ducts as a surrogate for pancreatic position during free‐breathing.^(19)^ These studies concluded that interfractional positional variation was not negligible and that interfractional reproducibility was higher at end‐exhalation then at end‐inhalation. One study, which used implanted fiducial markers,^(6)^ quantified the residual uncertainty in five patients on kilovoltage images gated in the end‐expiration phase during free‐breathing. In that study, the absolute value of the mean additional shifts observed after alignment to bony anatomy in the RL, AP, and IS directions was 0.18 cm, 0.16 cm, and 0.41 cm, respectively. The absolute maximum shifts in the RL, AP, and IS directions were 1.3 cm, 0.9 cm, and 1.9 cm, respectively. These data are calculated comparing gated images with simulation digitally reconstructed radiographies. Our data, obtained with respect to the first MV‐CBCT, showed similar findings along RL (1.51 cm) and AP (0.93 cm) directions, with a smaller value (1.05 cm) along the IS direction.

The interfractional shift can be associated with the fluctuation in the average position of the respiratory motion,^(20)^ as day‐to‐day breathing patterns can be quite variable. Accordingly, the largest additional shift to fiducials found in the IS direction is most likely related to variation in daily baseline diaphragm position.^(6)^ Moreover, the interfraction uncertainty we observed could be partially due to gastrointestinal motion, bowel gas, or stomach filling. Patient respiration training and/or patient preparation protocol could reduce this uncertainty.

Despite many studies reporting pancreatic intrafraction motion, and recently some studies analyzing interfraction reproducibility, to our knowledge only Shiinoki et al.^(19)^ and Whitfield et al.^(10)^ measured both intra‐ and interfraction displacements. In agreement with these studies, our findings suggest that after bony alignment in free‐breathing treatments, the magnitude of interfraction residual uncertainty requires additional margins, which may result of the same magnitude of the margins required to account for the respiratory excursion. A limitation of our study is that we evaluate interfraction residual errors with respect to the MV‐CBCT acquired during the first section instead of using the CT simulation, so that we were only able to correctly calculate $\Sigma_{\text{res}}$. According to Whitfield and colleagues,^(10)^ the same $\Sigma_{\text{res}}$ has been used as the best estimation of the preparation component of the residual error $\Sigma_{\text{res}}$.

Considering that in our findings MIFE and the residual errors were uncorrelated, they can be linearly added. In order to obtain patient specific margins, the additional margin can be reduced to half of MIFE if the respiratory phase is known.^(13)^ Whitfield et al.^(10)^ calculated treatment margins using a value for MIFE covering 95% of group intrafraction peak‐to‐peak motion. Consistently, this approach produced greater margin than those we obtained for each patient by using 4D CT. In fact, we obtained a good agreement in RL and AP directions, but a clear reduction along IS direction, where the respiratory motion is greater. However, due to day‐to‐day MIFE amplitude changes, intrapatient MIFE variation should be analyzed and patient mean amplitude evaluation from more cycles would be preferable with respect to evaluation on single 4D CT.^(21)^ As in our study, to minimize these effects, breath coaching should be strongly recommended before 4D CT acquisition. In fact, it was demonstrated^(22)^ that respiratory training (with visual and audio feedback when available) improves the reproducibility of the breathing pattern.

Our study demonstrated that, after bone alignment and using personalized margin, it is possible to perform conformal free‐breathing irradiation of locally advanced nonoperable adenocarcinoma of the head of the pancreas. In fact, at high doses (45 Gy plus a boost of 9 Gy), the application of the CONKISS technique permitted the fulfillment of the tolerance level recommended by NCCN guidelines, with the exception of the maximal doses to small bowel in two patients. In these patients, a lower dose might be prescribed (e.g., a boost of 7.2 Gy), according to NCCN guidelines, which recommend doses in the range of 50–54 Gy for unresectable disease.

Finally, the large deformation occurring in pancreatic motion could potentially limit the application of conformal free‐breathing irradiation. The motion of tumor borders rather than a single fiducial could be most important when defining PTV and could potentially lead to marginal misses in conformal treatment. However, the tumors spent the majority of time toward the exhale portion of the breathing cycle, whereas the time spent at the opposed extreme of motion was only a small fraction (<5%).^(5)^ Since we calculated the dose distribution at the end of expiration, the potential target underdosage due to deformation occurring in the opposed extreme of motion is minimized. On the contrary, any underestimation of interfraction uncertainties could produce target underdosage in the whole breathing cycle.

## V. CONCLUSIONS

Despite 4D irradiation being considered the optimal treatment in movable targets, such approach is technically — and resource — demanding, and conformal free‐breathing irradiation is still an option to treat pancreatic cancer. However, in free‐breathing irradiation, the use of personalized margins which properly include intrafraction motion should be recommended. Their assessment requires 4D methods only during simulation CT, and therefore this approach does not require additional technical/time resources during irradiation. Moreover, the interfractional residual uncertainty needs to be estimated and properly added to CTV to obtain the PTV. Using these margins, CONKISS technique allowed for the delivery of 45 Gy plus a 9 Gy boost, as well as obtaining satisfactory dose distribution according to NCCN recommendation, showing that conformal free‐breathing irradiation can be adopted in the radiotherapy departments with limited resources.
